# The Hospital School from the Health Professionals’ Perspective: Roles and Collaboration

**DOI:** 10.5334/cie.273

**Published:** 2026-03-16

**Authors:** Francesca Maria Dagnino, Giovanni Paolo Caruso, Edoardo Dalla Mutta, Chiara Fante, Vincenza Benigno

**Affiliations:** 1 Consiglio Nazionale delle Ricerche –Istituto per le Tecnologie Didattiche, Italy; 2 University of Genova –Department of Foreign Languages and Cultures, Italy

**Keywords:** Hospital School, Hospital Teachers, Interprofessional collaboration, Healthcare professionals

## Abstract

The paper explores health professionals’ (HPs) perceptions of the Hospital School (HS) service, focusing on its role within the care process and on interprofessional implications. The importance of HS in ensuring educational continuity for hospitalized children is widely recognised, however, studies show that teachers sometimes feel their role is not acknowledged by HPs and that the quality of interprofessional interactions strongly affects teachers’ work and well-being. Using a qualitative approach, individual interviews followed by a focus group, this study investigated HPs’ point of view by asking them about 1) their perception of the HS service and teachers, 2) their interactions with HS teachers, and 3) how their collaboration might be supported. Results indicate that the role of HS is perceived in a variety of manners, and relationships with teachers are often driven by individual rather than organizational-mediated practices. Solutions proposed to better integrate HS within the hospital and strengthen collaboration between teachers and healthcare professionals highlight the need for both institutions to actively promote structured opportunities for reciprocal knowledge building, and to introduce coordinating figures that connect teachers with each other and with healthcare staff. HPs’ responses in this study reflect challenges reported in the literature regarding inter-professional collaboration among HPs and suggest that some of the strategies developed to improve collaboration within healthcare teams could be adapted to strengthen cooperation between HPs and HS teachers.

## Introduction

Chronic or acute medical conditions, including mental health disorders, that necessitate patients extended or recurrent hospitalization, or even permanence at home, constitute profound disruptions in the developmental trajectories of children and adolescents (Fardell et al., 2023; Brady et al., 2021). Beyond the physical and psychological distress associated with illness and its treatment, young people in this situation experience an abrupt interruption of their academic and social life (Jay et al. 2025). Indeed, these conditions are often associated with a range of negative impacts: periods of discontinuance of school attendance; reduction of engagement and motivation to learn (Lum et al., 2017); lower academic achievements (Forrest et al., 2011; Wikel & Markelz, 2023); lower sense of school belonging (Tomberli & Ciucci, 2021); and problems at school re-entry (Shaw & McCabe, 2008; Capurso et al., 2025). Another critical issue concerns the breakage of relationships with peers (Forrest et al., 2011), partially linked with the interruption of a regular school life. All these changes impact the children’s psychological and emotional wellbeing (Brady et al., 2021).

Hospital School (HS) is a service aimed at guaranteeing the right to education for hospitalized students, who, for longer or shorter periods, cannot attend school regularly. This service has been established and supported at different times in many countries in Europe and around the world; its organisation and nature often differ in response to national regulations (LeHo Project, 2015). HS makes it possible to mitigate some of the before-mentioned issues (Caggiano et al., 2021); children can benefit from personalized learning interventions tailored to their specific needs, whose impact goes beyond educational continuity. HS also contributes to maintain a sense of normality in the children’s life and represents a bridge to life outside of the hospital (Benigno et al. 2017). HS ensures social interactions with teachers, educators and, where possible, with other children, counteracting the sense of isolation that can result from hospitalization (Capurso et al., 2021; Sullivan et al., 2001). Furthermore, HS represents an essential part of the overall care process, rather than a secondary element, as when planning the learning intervention, guaranteeing the well-being of the young patient is taken into account (Lum et al., 2017). The distinguishing feature of hospital schooling is that it takes place in a context in which the primary focus is the children’s care. Hospital teachers tend to perceive themselves, and are perceived by health professionals (HPs), as ‘guests’ in an environment governed by schedules, rules and priorities that differ from those to which they are accustomed (Hen & Gilan-Shochat, 2022). While working in clinical environments, hospital teachers must also remain accountable to educational authorities, thereby navigating a dual system that integrates educational and healthcare demands (Field & Lewis, 2025). While HPs have started to recognize the role of educational continuity in therapy support (Di Padova et al., 2024), ‘cohabitation’ is not always easy; it represents a further challenge that is associated with those linked to the relationships with children suffering from medical conditions and their families (Benigno & Fante, 2020; Steinke et al., 2016). Indeed, the study by Benigno and Fante (2020) highlights how hospital teachers are subject to stress due to the working context and relationships with healthcare professionals. A central issue is the lack of recognition of their role in the childcare journey, reflected in limited inclusion in formal staff meetings and extemporaneous contact with care staff. Moreover, the study highlights that a key source of stress is the work fragmentation linked to the frequent interruption of lessons due to medical visits and treatments, as well as time and contextual constraints. Coordination with health care professionals is also recognized as a challenge by hospital teachers in Spain (Jiliberto & Zárate Alva, 2025). In this regard, a crucial aspect of the hospital teacher’s work is having adequate knowledge of their students’ clinical conditions, which can only be ensured through good communication with the medical staff. Only through appropriate assessment of the potential limitations students face on a daily basis due to their illnesses, HS can provide effective education; there is a need for consideration of the short- and long-term effects of the child’s physical and psychological condition on their learning potential (Hopkins et al., 2014; Keehan, 2021).

### Hospital School Services in Italy

In Italy, which is the focus of this study, HS services were initially introduced by individual hospitals as early as the 1920s. Subsequently, HS sections for primary education began to spread in the 1950s. However, HS was officially established in 1986, under the Ministerial Directive No. 291. Currently, HS is managed by the Ministry of Education and Merit; it covers all school levels and is active in most wards and paediatric hospitals in the country. A series of guidelines for HS have been issued, including both organizational and educational aspects, by the Ministry (MIUR, 2019). At local level, HS is managed by a Central Hub School, which is responsible for coordination of teachers’ activities. Oversight of HS at regional level is handled by a technical committee that includes representation from the Hub School, the director of the regional school authority, and the manager of the local health authority. A specific policy document indicates the responsibilities and activities of each body involved, with the purpose of promoting the well-being of hospitalized students (MIUR, 2019).

In the past decades, two HS training courses were held in Italy in collaboration with the Ministry of Education with the aim of equipping hospital teachers with the necessary tools and competencies to carry out their work in hospital settings (Benigno & Vallarino, 2009; Benigno et al. 2018). These courses were a tangible response to the lack of teacher training for this specific professional context (LeHo Project, 2015; McNamara, 2024). The courses involved most of hospital teachers nation-wide and were aimed at promoting skills across organizational, relational, psycho-pedagogical and technological dimensions.

A comprehensive survey involving 95% of hospital educators (Benigno et al., 2017) showed that HSs are breaking away from traditional schools with their well-defined routines, spaces, roles, and rules, transforming themselves into dynamic and flexible schools capable of adapting to the needs of context and individual students, adopting flexible strategies and operating models while maintaining their institutional role. Benigno and colleagues (2017) note that physical spaces available are often improvised and non-permanent; although some hospitals do have fixed equipped classrooms, many teachers find they have to adapt to using patient rooms, meeting rooms, or even waiting rooms. The same survey states that 60% of teachers have access to a dedicated educational space, while others use non-exclusive or alternative environments; moreover, 54% of teachers state that they often work in multiple wards (Benigno et al., 2017). These results confirm the common divergence between traditional schooling typically conducted in fixed spaces, and the fluctuation that characterises HS, where it is the school that moves toward the students and not vice versa. With regard to relationships between HS teachers and HPs, Benigno and colleagues (2017) reported that there are frequent contacts but of a predominantly informal nature, meaning that they do not occur in structured team meetings. These contacts are aimed at exchanging information about the child’s conditions only when deemed necessary. This suggests that teachers are not fully integrated into the hospital context, and that there is a lack of a true multidisciplinary approach in the comprehensive management of young patients. However, frequent informal contacts indicate a recognition of the teacher as an active ‘presence’ in the care context.

In the light of the attention towards interprofessional collaboration among health professionals (World Health Organization [WHO], 2010) and the impact of collaboration among professionals on the quality of patients and health systems outcomes (Reeves et al., 2017), it is worth exploring the interprofessional collaboration among health and educational professionals specifically in paediatrics wards or hospitals. Despite the importance attributed to a multidisciplinary approach involving non-healthcare professionals (Tondi, 2022), this issue is scarcely explored in studies, especially from the HPs’ perspective. Di Padova et al. (2024) address this topic by introducing the concept of Educating Community and briefly mention that HPs can contribute to the work of teachers. On the other hand, Kanizsa (2024) points out that teachers are often excluded from multidisciplinary teams because HPs (and sometimes parents) tend to consider illness as prevailing over the other important aspects of a child’s life, thereby losing a truly holistic view of the child. Another aspect that the author underlines is how the different professional backgrounds of HPs and teachers can lead to different approaches and languages (Kanizsa, 2024). Given the framework outlined and the lack of research investigating the point of view of health workers, the present study aims to go a step further, by closely examining HPs’ perception of the HS service and its integration in the hospital context. Moreover, we sought to explore solutions with HPs to strengthen reciprocal knowledge and collaboration. We adopted an exploratory approach to the topic, following open-ended investigation design guided by participants’ perspectives (Busetto et al., 2020). This enabled the collection of nuanced individual accounts, including both facilitators and challenges (Willig & Rogers, 2017).

Specifically, the research questions (RQs) we address in the study are as follows:

1. How are HS and HS teachers perceived by health professionals?
2. How do health professionals interact with HS teachers?
3. How can collaboration between teachers and health professionals be supported?

## Method

### Context of the Study

The study was carried out at the Giannina Gaslini Hospital, a scientific Institute for Hospitalization and Treatment based in Genoa, northern Italy. The hospital addresses a comprehensive spectrum of paediatric and surgical specialties at the highest level, and has a staff of about 2000 employees, including HPs, and administrative and technical personnel. The hospital has a national and international profile; it counts about 30,000 hospitalizations and 700,000 outpatient treatments annually; these include 42% of patients from outside the hospital’s region, not to mention a total of about 200 patients from over 70 countries in the world (https://www.gaslini.org/istituto-gaslini/chi-siamo/listituzione/). This medical institute also has a strong tradition in training, managing two university courses and doctoral programs. HS has been present there since 1976 and now covers all school levels (upper secondary school opened in 2003). Currently there are about 50 teachers working at the hospital. Kindergarten and primary teachers are often assigned to single wards, while secondary teachers move across wards, given the specificity of the subjects they teach. Teachers are mostly present during the morning, as per mainstream schooling in Italy, and usually carry out lessons at the students’ bedside or in playrooms located inside the wards. Kindergarten teachers work also with older students, involving them in playful activities.

### Study Design

The study was exploratory in nature and employed a qualitative methodology with two main data collection methods: a sequence of in-depth, individual online semi-structured interviews (Adams, 2015) and a semi-structured focus group (Acocella, 2005). The interviews were carried out with the aim of answering RQ1 and RQ2, i.e. to explore the perception of HS and interaction between HP staff and HS teachers. The key points that emerged from the interviews provided the basis for the focus group session. The focus group stage was designed and implemented with the aim of answering RQ3 regarding useful actions to improve collaboration between the hospital and HS. The focus group method was preferred to interview here since it makes it possible to collect new ideas by leveraging group dynamics and synergies (Acocella, 2005).

Participants were recruited through convenience sampling. Two different groups of HPs were involved in the two different phases, with just one professional participating in both. Participants’ involvement was mediated by the hospital. A call for availability for a one-hour interview was sent to the hospital, which in turn conveyed the call to physicians, nurses and psychologists. The same process was followed for the focus group, but in this case the hospital selected professionals covering positions of responsibility, such as medical heads of wards and nursing coordinators.

### Methods

The semi-structured interview was specifically developed to meet the objectives of the study. The interviewers followed a specific protocol that was developed following the recommendations of Jacob and Furgerson (2012). The protocol included relevant information about the study, open-ended questions to be addressed (but with the freedom to explore contents emerging during the interview), and, finally, some suggestions for conclusive remarks. The interview protocol is available in Appendix A. The questions it contains were developed by the research team on the basis of results from a previous study carried out at national level. There, the relationship between HS teachers and HPs was raised as a critical factor (Benigno et al, 2017). Before the interviews, the questions were piloted with a colleague not involved in the research. The stimuli employed in the focus group were developed based on findings from the interviews and from previous investigations. These were designed to prompt HPs to propose solutions to the specific issues identified during the interview phase:

1. How can the hospital institution and health personnel support the work of hospital teachers?
2. What actions could be taken to strengthen reciprocal awareness of the two institutions?
3. How could a practice of exchanging patient/student information between health personnel and teachers be created?

### Participants

- Interviews: six HPs, including three nurse coordinators, one nurse, one physician and one psychologist. All the participants were women with a mean professional experience in paediatrics of 27.5 years (min 9 – max 37).
- Focus group: seven HPs, including three nurse coordinators, two physicians and two psychologists. The group comprised two men and five women with a mean professional experience in paediatrics of 18.5 years (min 4 – max 41).

### Procedure

The interviews were carried out online by four researchers following the aforementioned protocol (see Appendix A). Before being interviewed, participants gave informed consent to the recording, processing, and use of the material collected. All the interview sessions were then recorded and transcribed ‘verbatim’ by a member of the research team.

The single focus group was carried out face to face by three researchers in a single session that lasted approximately 1.5 hours; the three respectively played the role of moderator, co-moderator and recorder. Another researcher attended the session remotely, supporting their recorder colleague. Initially, the moderator introduced the session topic by referring to the results of the interviews and research previously carried out on the main stress factors in HS teachers’ professional activity. As was the case for the interviews, participants gave their informed consents beforehand. The recordings of the focus group were transcribed ‘verbatim’ by a member of the research team.

### Data Analysis

Both interview and focus group transcripts were coded by the four researchers using a bottom-up, inductive approach. The Reflective Thematic Analysis (RTA) framework (Braun & Clarke, 2022) was adopted, as it aligned with our goal of interpreting, synthesizing, and reporting themes that reflect participants’ experiences. Data analysis was conducted inductively and collaboratively: codes and segments of interest were discussed among the researchers, generating shared reflections and contributing to a richer and more nuanced interpretation of the dataset (Braun & Clarke, 2019). The researchers first familiarized themselves with the data by reading the interview transcripts as a single text, and then proceeded to code them independently. The analysis was semantic, and codes were drawn from the explicit meanings identified in the data. The individual coding phase was carried out manually using the comments function in Microsoft Word. Coders generated individual code sets and then these were merged; interpretative differences were addressed in such a way as to maintain the richness of multiple coding but avoiding conflation of similar codes. Finally, a codebook was prepared as a preliminary step to theme generation. Codes were aggregated under themes and subthemes on the basis of shared meanings through a recursive process. The same process was followed for the transcriptions of the focus group. The final codebook with codes, themes, and subthemes, and exemplar definitions is reported in Appendix B.

## Results

The results of the interviews and focus group discussions relating to the three research questions are now presented. Thematic analysis of the interviews and the focus group was conducted separately and resulted in development of two distinct codebooks. As stated, the stimuli for the focus group stemmed from the interviews. Despite the use of different prompts, several overlapping themes emerged across both data sources. Although the focus group was oriented toward identifying solutions (RQ3), it also reinforced the interview findings, contributing to comparison of results and strengthening of overall thematic coherence. Specifically, HPs often returned to issues initially raised in the interviews, exploring them in greater depth. Table 1 presents an overview of the themes, indicating whether they were identified in the interviews, the focus group, or both.

**Table 1:** Mapping of the themes in the study interviews and focus group.


RQ1 – HOW ARE HS AND HS TEACHERS PERCEIVED BY HEALTH PROFESSIONALS?		

THEMES	INTERVIEWS	FOCUS GROUP

1. The function of the hospital school	present	

2. HS integration in the care process	present	present

3. Perceived teachers’ difficulties in hospitals	present	present

**RQ2 – HOW DO HEALTH PROFESSIONALS INTERACT WITH HS TEACHERS?**		

4. Relationships between health professionals & teachers	present	present

**RQ3 – HOW CAN THE COLLABORATION BETWEEN TEACHERS AND HEALTH PROFESSIONALS BE SUPPORTED?**		

5. Institutional and personal support for HS teachers’ work		present

6. Solutions to foster reciprocal knowledge		present

7. Solutions to foster communication		present


In Tables B1 and B2 (see Appendix B), codebooks with codes, themes and exemplar definitions are available.

### RQ1 – How are HS and HS teachers perceived by health professionals?

#### Theme one: The function of the hospital school

Health professionals spontaneously adopt positive terms to define HS, confirming a perception of the service as important to the wellbeing of hospitalized children and youth. They refer to the HS as a service that guarantees a sense of normality to hospitalized students, and that anchors the students to the outside world (see Table B1, Appendix B). This ensures the right to education and educational continuity, which act as a bridge for school re-entry, aspects that are extremely important both for students and their families, even though this might seem secondary compared to the individual’s health condition:

“*Then I believe that the hospital school represents an institutional response to two fundamental rights of children and adolescents, more generally of school-age children, which are the right to study and also the right to health, and the hospital school service allows minors who are temporarily or chronically ill […] to avoid interrupting their course of study”*.

In addition to pedagogical aspects, HS is considered an emotional and relational resource. Indeed, it is recognized as invigorating the healthy part of the student, counteracting loneliness and apathy, and fostering socialisation within the hospital:

*“Somehow, they [hospitalized students] get depressed, they become apathetic, they don’t feel like reading, or they are always on their phones etc. The fact is that there is, nevertheless, a commitment that is a life commitment for them. Well, this is fundamental here: I consider it very important.”*

HS teachers are often identified as an affective figure, and the stimulating presence of the HS is recognized as a contribution towards increasing response to medical treatments:

*“In addition, let’s say that a greater presence of the patient, of the young person, through continuous educational activity, etc., also makes them more receptive to all the medical treatments”*.

#### Theme two: HS integration in the care process

Despite the recognition of the importance of HS teachers’ role in the patient’s clinical pathway, HPs engaged in the interviews expressed different viewpoints regarding the integration of HS teachers in the care process. HS is seen by some HPs as complementary, by others as an integral part of the pathway (see Table B1, Appendix B). Nevertheless, teachers’ participation in staff meetings is seen as not feasible by some of the professionals, for a variety of reasons (including a general difficulty in holding staff meetings):

*“[…] including a teacher is not possible and I don’t even objectively see the benefit of it”*.

A shared opinion expressed in the focus group was that HS and hospitals are not well integrated and, therefore, the HS is not part of the care program in all the wards:

*“So, if we speak of a hospital institution, it is clear that in reality this is not the case: they are two realities that intersect and sometimes even conflict with respect to those… let’s call them urgencies or difficulties […]”*.

#### Theme three: Perceived difficulties of teachers in the hospital context

In the interviews, HPs report having identified several difficulties teachers face: problems with logistics (related to moving between wards or to the absence of dedicated spaces); critical issues related to coming into contact with the student’s illness; integration with healthcare personnel; and emotional/relational and privacy issues (see Table B1, Appendix B). Regarding relations with their colleagues, HPs report the lack of attention to – or respect for – the teachers’ work of some HPs:

*“Other difficulties they certainly find: maybe they arrive, they start the lesson, the doctors arrive, and they are forced to interrupt the lesson. I don’t find that fair either”*.

In the focus group, HPs highlighted some similar stress factors shared by HS teachers and hospital staff. According to one of the participants, the functioning of the hospital is at the root of the difficulties of both categories, including work fragmentation, the absence of adequate spaces, and communication issues:

*“It is not so much a difficulty in communication between people, but the institution that builds blocks within which communication then becomes difficult”*.

Fragmentation of teachers’ work is attributed to the fact that, in the hospital setting, diagnostic and therapeutic needs take precedence over educational ones. Moreover, there is a lack of support figures to facilitate communication with the other ‘actors’ (families, health professionals, etc.) due to a general paucity of figures such as occupational therapists and educators (see Table B2, Appendix B).

One key point is the issue of privacy. According to the interview respondents, privacy regulation significantly affects the possibility to share information about patients. Nevertheless, there are those who see no impediment to sharing information about the child’s ‘functioning’ (the cognitive implications of the disease or treatments) without sharing the diagnosis:

*“It is not as important for the teacher to know the name and surname and mere diagnosis of what the child has, as what the pathology entails in carrying out of the learning activity”*.

Privacy issues were also mentioned during the focus group. Health professionals also recognized this as hindering communication. However, reference is made to the ‘boundaries’ of privacy, reflecting a similar approach to those expressed during the interviews; certain information concerning patient functioning could be usefully exchanged without incurring any violation:

*“[…] that information goes beyond privacy is part of the treatment […], rather what are the problems, what are the weaknesses, what channels can there be for a greater possibility of involvement. We, who have been living with them [patients] from the beginning, can give information to them [teachers] and they should transfer to us what the attitude was…”*.

On the contrary, one professional expressed doubt about the soundness of sharing information, stressing the perception of teachers and HS as a source of normality:

*“School has its own specificity, which is the fact that it also carries a dimension of ‘normality’, i.e. [in ]developmental [terms], in which it is also important that the teacher does not approach the child, to the youngster, thinking that he or she is at risk of suicide, has a serious incurable illness, namely that there is also a space of freedom”*.

A further obstacle to communication that emerged is the large number of teachers working with the same students (especially secondary school students).

### RQ2 – How do health professionals interact with HS teachers?

#### Theme four: Relations between health professionals and teachers

As in all human relationships, multiple factors influence the relations between HPs and HS teachers. Firstly, the teachers’ personal characteristics and behaviours: HPs appreciate those who have highly attentive approaches and are respectful of the hospitalized students and their families, and with the staff as well:

*“They are really two special teachers, two people, well… beyond the fact that they are very polite and very kind […]”*.

Teachers’ flexibility in carrying out their work is considered important:

*“Also, working in [name of the ward] requires an enormous amount of flexibility because not all activities are suitable for everyone and because the patients have this very fluctuating trend…Sometimes the teacher gets an idea ‘OK I go to patient X and I do this’ and they come here and see that that can’t be done, you absolutely have to change”*.

In addition, the frequency of interaction significantly impacts the quality of relationships (see Table B1, Appendix B). When teachers are permanently assigned to a specific ward, their relationships with HPs transcend formal boundaries. Conversely, interactions with secondary school teachers, who rotate between wards, typically remain formal and are not always smooth or harmonious.

Anyway, from the interviews there is an evident lack of integration between HS and the hospital. Most HPs have scant information about the functioning of the HS service. Care activities take priority over teaching activities, and collaboration between physicians and teachers is limited. What’s more, the concurrence of several teachers in the ward is seen as disruptive:

*“In the morning, we receive teachers, maybe from high school, of other subjects that my teacher [the teacher assigned to the ward] does not teach. I get two or three in the morning, ehh a bit of…a bit of…in short, they create a bit of chaos for me”*.

Nevertheless, teachers are mentioned as a valuable source of information:

*“[…] because through them you also understand how a child is feeling, if they tell you ‘Look, we managed to study, we managed to do this, we talked, we did…’, [the patient] is better; also clinically this corresponds to a clinical improvement”*.

In line with the findings from the interviews, during the focus group professionals recognize the lack of knowledge of how HS works (see Table B2, Appendix B). This is reflected in the scarce information about the organization of HS (e.g., different teachers for different school levels, lesson schedules). This is attributed to a lack of communication between the two institutions:

“*Teachers arrive just like that. We don’t have a schedule, we don’t know, and sometimes they arrive and don’t even find the patients”*.

### RQ3 – How can collaboration between teachers and health professionals be supported?

The focus group was intended to gather suggestions on how to support the teachers’ work and improve integration between HS and the hospital. Through transcription analysis, three themes were identified (see Table B2, Appendix B).

#### Theme five: Solutions to support the HS teachers’ work

Several solutions were proposed to support the teachers’ work.

At the institutional level, healthcare professionals emphasize the importance of the hospital’s commitment to supporting the work of teachers, and refer to the presentation of HS as an integral part of the care process:

*“Perhaps an important factor is that of the presentation of the school service to patients, in the sense that it is often presented as part of the care pathway; if this becomes an element of team culture, the teacher would probably integrate in a different way”*.

According to the participants, this is not always possible since there are differences between wards; in this light, professionals highlight the importance of the identification of solutions tailored to the needs of single wards given the differences in patient population (type of hospital stay, age, etc.) and objectives (e.g. diagnostics, therapy, rehabilitation, etc.).

Focus group participants supported a general call for commitment to adopt a more integrated vision so as to overcome the fragmentation of hospital work.

In line with the problems explored, HPs suggest identifying liaison figures (e.g., psychologists, nursing coordinators, a single teacher assigned to the ward) to support the relationship with hospital teachers.

#### Theme six: Solutions to foster reciprocal knowledge

On this point, HPs suggest possible solutions for adoption by both the hospital and the HS. They range from training actions to formal and informal opportunities for knowledge and personal acquaintance. Specifically, a proposal was made to create training paths for teachers about specific wards, but at the same time some expressed the need to familiarize themselves with HS functioning.

*“[…] the training of teachers with respect to the situations they face, because a psychiatry ward is different from a bone marrow transplant ward, where patients are isolated, where in any case teachers are often struggling… it is probably also a protective factor for teachers to be trained in the various situations they face, which are so varied that they bring different needs and are certainly also problematic in different relationships”*.

To foster personal acquaintance with hospital teachers, HPs highlight the need for formal meetings at the beginning of the year to get to know each other:

*“[…] at least introduce us… I mean, we really don’t have much [acquaintance]… at the beginning of the school year… it would also take a meeting, something where we start a path at least for the school year, get to know each other…”*.

#### Theme seven: Solutions to foster communication

Health professionals suggested scheduling periodic discussion meetings, and expressed the need to share objectives while respecting roles:

“*Maybe sharing, I’m thinking, is the sharing of objectives as well as information, right? In the sense that beyond everything, maybe if there is a plan for the individual, each one for their skills and for their part. This is part of a process in which objectives, perhaps, are shared”*.

Finally, suggestion is made to appoint a coordinating figure among the teachers, so as to have a single contact person:

*“So, there is an organizational problem there, a problem of resources, of personnel, and also a problem of thinking of a figure who coordinates a little bit the school activity on that ward and therefore a figure who is the representative…”*.

## Discussion

The study presented in this article sought to explore the role of HSs and how they are perceived by healthcare professionals. Regarding the first research question, three significant themes emerge: (1) the role of the HS; (2) HS integration in the care process; and (3) perceived difficulties teachers experience in the hospital context

The interviews conducted with hospital staff as part of the study show that HSs represent a concrete implementation of the universal right to education (Art. 26, The United Nations, 1948). They ensure hospitalized children have access to learning and social interaction, as also demanded by the European Charter of Rights for Children in Hospital (EACH, 2002). Article Seven of the charter, affirms every child’s right to education, play, and recreation appropriate to their age and condition, with specific attention to maintaining their educational progress. In relation to the integration of HS into the care process (Theme two), one of the most critical aspects already highlighted in previous studies emerges (Benigno et al., 2017): not all HPs agree on the actual need to include teachers in the multiprofessional team. Difficulty in interacting with medical staff during structured and scheduled sessions, however, may reflect a critical aspect of the teaching profession, especially if related to a lack of recognition of their role within the ward. This hypothesis appears to be confirmed by other data collected from the survey that Benigno & Fante (2020) conducted into the stressful elements associated with their profession. Although the role of teachers is formally recognized, there is a lack of structured spaces for discussion allowing dialogue and mutual understanding between healthcare professionals and teachers. Consequently, teachers, despite being key figures in addressing the educational and relational needs of minors, are often perceived as extraneous to the care process in the strictest sense. Nevertheless, certain information about a student’s health status can be instrumental in effectively tailoring educational experiences to individual needs (Keehan, 2021). A particularly critical issue that emerged from the reported study concerns the management of privacy in the exchange of information on students’ health that takes place between healthcare professionals and teachers. Opinions among healthcare professionals are divided: some believe that sharing diagnoses would bring no benefit to the teacher’s work, while others consider it would be useful for guiding and adapting teaching. The issue of privacy is therefore a complex and sensitive area, requiring greater awareness from both an ethical and regulatory perspective, with reference to formal agreements and current protocols. The official inclusion of teachers within the multidisciplinary team, through an institutionally recognized agreement, could facilitate more transparent and effective management of clinical information relevant to the educational process. However, as Kanizsa (2024) points out, one of the main difficulties in integrating teachers into healthcare teams is consideration of the HS as a merely recreational activity. Added to this is a vision of illness as an all-encompassing condition, which tends to obliterate every other dimension of the child’s person: it eclipses their identity, their educational needs, and the continuity of their development, even during hospitalization. Finally, the third theme expresses heightened awareness among healthcare professionals of the challenges teachers face in the hospital setting, which has little to do with traditional schooling. This awareness could be a starting point for creating spaces for sharing and mutual support, even considering the creation of Communities of Practice (CoPs) among professionals who care for the broader well-being of vulnerable children (Field & Lewis, 2025).

Regarding the interpersonal relationships between HPs and HS teachers, results show that these are often grounded on individual initiatives and are influenced by personal characteristics and opportunities. Overall, it is evident that HS and hospitals as organizations do not facilitate reciprocal knowledge and collaboration, and that most of the healthcare professionals involved in the study are not familiar with how HS works. Difficulties in relations between teachers and HPs can be observed through the lens of interprofessional collaboration in care contexts. Looking at the literature in the field, which mostly regards different health professional groups, it is possible to identify several barriers, some of which are also evident in our findings. In an umbrella review by Wei and colleagues (2022), authors categorize barriers at three levels: organization, team and individual. At organizational level, our results also point to the lack of communication at system level, scarcity of guidance and routines, and the presence of competing priorities. More specifically, medical procedures are considered as overriding educational activities, and this leads to frequent interruptions of HS lessons, causing fragmentation in the teachers’ work. At individual level, the barriers reported in the literature regard interpersonal communication and relationships. According to interviewees in this study, relationships with teachers are not always smooth due to the tendency, especially of the newcomers or those who move across wards, to have limited interaction with healthcare staff. Nevertheless, in our interviews we also detected a subtle ‘closure’ on the part of at least one professional. Certainly, as highlighted by Benigno and colleagues (2017), smooth communication with medical staff requires good interpersonal skills and openness on the teachers’ part.

Concerning possible solutions to support collaboration, it is important to emphasize that the themes identified in the study refer exclusively to data collected from the focus group. The primary objective of that focus group was precisely to gather suggestions regarding possible solutions for enhancing mutual understanding and collaboration between healthcare and school professionals. The adoption of the focus group, conducted following the individual interviews, played a pivotal role in sparking ideas for co-constructing practical strategies, that could enrich the value and meaning of the teachers’ work. Three main themes emerged here: Solutions to support the HS teachers’ work; Solutions to foster reciprocal knowledge; and Solutions to foster communication.

The indications proposed by HPs tend towards integrating and complementing the respective teams and creating formal moments of acquaintanceship and communication. Once again, these suggestions from HPs reflect the findings in the literature on interprofessional collaboration in healthcare contexts and refer to organization policies, to professionals and teachers training and to individual actions. Indeed, organizations are the wheel that facilitates interprofessional collaboration in terms of culture, structure and support (Wei et al., 2022). The situation we analysed also entails interorganizational collaboration (HS and hospital), something that converges with the results reported by Karam and colleagues (2018). These identified, among other factors, communication, mutual acquaintanceship, and patient-centeredness as important aspects for both interprofessional and interorganizational collaboration. As far as communication is concerned, the role of the organization is central to setting up protocols and agreement to facilitate it (D’Amour et al., 2008 cited in Karam et al., 2018). Shared goals and visions are cited as aspects that may facilitate interprofessional and interorganizational collaboration (Karam et al., 2018). In our findings, there was a call for a more integrated vision at the hospital level to overcome the fragmentation of work; one participant referred clearly to the opportunity of sharing goals with teachers. HPs also proposed training initiatives aimed at enhancing mutual understanding; however, no one mentioned training actions targeting interprofessional collaboration. Interestingly, the international literature strongly advocates for reinforcing interdisciplinary training, both during university education and in professional practice, as a key strategy to improve collaboration in healthcare contexts and between HPs and teachers (Hillier et al., 2010; Supper et al., 2015).

At an individual level, HPs highlighted the importance of building relationships of trust and mutual respect with teachers, in line with previous evidence (Wei et al., 2022). This process seems easier with teachers permanently assigned to single wards, but a reciprocal effort should also be made with those who move across wards. A liaison or coordinating figure are advocated by several HPs.

The proposed solutions reflect the existence of well-recognised actions which are not applied, at least in the context of our investigation. This might be due to a number of reasons, ranging from the lack of awareness of the difficulties that teachers face in relationships with HPs to the lack of recognition of hospital schooling as part of the care pathway at organizational level.

## Conclusion

The Hospital School (HS) is an essential service within children’s hospitals and wards; it guarantees educational continuity for students who are prevented from attending school due to their health conditions (Capurso et al., 2025). Despite the recognized importance of the service, the integration of HS in hospitals still needs supporting actions, as revealed both by previous research (Benigno et al. 2017; Jiliberto & Zárate Alva, 2025) and the results of this study. The concept of ‘educating community’ proposed by Di Padova and colleagues (2024) does not appear to be concretely achieved, at least in our study. The study’s findings advance understanding in a previously underexplored area of research, hospital professionals’ perspective on HS. It offers novel insights that can inform targeted strategies to address the critical issues identified. Building collaborative routines between medical staff and schools requires profound organizational changes and the implementation of different modes of interaction between stakeholders. The recognized goals of this collaborative relationship include sharing information on individual cases, working with families, monitoring response to interventions, and conducting interdisciplinary interventions (Shaw & Brown, 2011). Our findings reflect the advisability of extending some of the solutions already explored for enhancing interprofessional collaboration in healthcare to support health professionals-HS teachers’ collaboration. Redefining and reimagining the hospital space from a traditional place of care to a multidisciplinary space dedicated to health, development, and learning represents the fundamental objective of HSs and health services in general (Hopkins et al., 2014; St Leger, 2012). From this perspective, teachers have a privileged and irreplaceable role in maintaining a sense of continuity and ‘normalcy’ for young patients.

### Limits of the Study

The main limit of the study is that it involved a limited number of HPs in a single hospital. This failed to account for the differences in the functioning of HS in other hospitals in Italy and elsewhere. As a small-scale qualitative study, we are not aiming for generalization of results. Our intention was to carry out the study and report it in such a way as to ensure its trustworthiness. We provided a detailed description of our own study context and of the methodological aspects involved, and we compared data from two sources, namely interviews and a focus group: readers can evaluate the scope for transferability to their context.

### Implications for Further Research

After this exploratory phase, a quantitative study involving a larger sample of HPs could help us to better understand integration of the HS in hospitals of different size and structure, and the parallels with interprofessional collaboration among HPs. We understand that the scope to tailor results in specific organizational contexts involving both school and hospital is essential.

## Additional Files

The additional files for this article can be found as follows:

10.5334/cie.273.s1 Supplementary File 1. Appendix A – Interview protocol.

10.5334/cie.273.s2 Supplementary File 2. Appendix B – Codebooks.

## References

[1] Acocella, I. (2005). L’uso dei focus groups nella ricerca sociale: vantaggi e svantaggi. Quaderni di Sociologia, 37. 10.4000/qds.1077

[2] Adams, W. C. (2015). Conducting Semi-Structured Interviews. In K. E. Newcomer, H. P. Hatry, & J. S. Wholey (Eds.), Handbook of Practical Program Evaluation. Wiley. 10.1002/9781119171386.ch19

[3] Benigno, V., & Fante, C. (2020). Hospital School Teachers’ Sense of Stress and Gratification: An Investigation of the Italian Context. Continuity in Education, 1(1), 37–47. 10.5334/cie.14 38774532 PMC11104412

[4] Benigno, V., Fante, C., & Caruso, G. P. (2017). Docenti in ospedale e a domicilio. L’esperienza di una scuola itinerante [Teachers in hospital and at home: the experience of a traveling school]. Franco Angeli.

[5] Benigno, V., Fante, C., Epifania, O., Caruso, G., & Ravicchio, F. (2018). A dynamic model for distance learning: Evaluation of an online course for hospital teachers’ professional development. Italian Journal of Educational Technology, 26(1), 90–103. 10.21125/iceri.2017.1312

[6] Benigno, V., & Vallarino, E. (2009). L’e-learning nella formazione dei docenti ospedalieri: il caso HSH@teacher. In N. Piave (Ed.), E-learning in sanità. Collana Nuove Frontiere Pedagogiche (pp. 63–81). Barbieri. ISBN: 978-88-7533-046-0.

[7] Brady, A. M., Deighton, J., & Stansfeld, S. (2021). Chronic illness in childhood and early adolescence: A longitudinal exploration of co-occurring mental illness. Development and Psychopathology, 33(3), 885–898. 10.1017/S0954579420000206 32362290

[8] Braun, V., & Clarke, V. (2019). Reflecting on reflexive thematic analysis. Qualitative Research in Sport, Exercise & Health, 11(4), 589–597. 10.1080/2159676x.2019.1628806

[9] Braun, V., & Clarke, V. (2022). Thematic analysis: A practical guide. SAGE. 10.53841/bpsqmip.2022.1.33.46

[10] Busetto, L., Wick, W., & Gumbinger, C. (2020). How to use and assess qualitative research methods. Neurological Research and Practice, 2(1), 1–10. 10.1186/s42466-020-00059-z 33324920 PMC7650082

[11] Caggiano, G., Brunetti, L. I. G., Ho, K., Piovani, A., & Quaranta, A. (2021). Hospital school program: The right to education for long-term care children. International Journal of Environmental Research and Public Health, 18(21), 11435. 10.3390/ijerph182111435 34769951 PMC8583304

[12] Capurso, M., di Castelbianco, F. B., & Di Renzo, M. (2021). “My life in the hospital”: Narratives of children with a medical condition. Continuity in Education, 2(1), 4–25 10.5334/cie.12 38774894 PMC11104414

[13] Capurso, M., Moracci, V., & Borsci, S. (2025). Pathways to school reentry for children and young people with a medical or mental health condition: An international Delphi Study. Continuity in Education, 6(1), 38–57. 10.5334/cie.159 40061057 PMC11887473

[14] D’Amour, D., Goulet, L., Labadie, J. F., San Martin-Rodriguez, L., & Pineault, R. (2008). A model and typology of collaboration between professionals in healthcare organizations. BMC Health Services Research, 8, 188. 10.1186/1472-6963-8-188 18803881 PMC2563002

[15] Di Padova, M., Pettoello-Mantovani, M., & Dipace, A. (2024). School in the hospital. The key role of an educating community. Global Paediatrics, 9, 100204. 10.1016/j.gpeds.2024.100204

[16] European Association for Children in Hospital. (2002). The EACH charter and annotations. EACH. https://www.each-for-sick-children.org

[17] Fardell, J. E., Hu, N., Wakefield, C. E., Marshall, G., Bell, J., Lingam, R., & Nassar, N. (2023). Impact of hospitalizations due to chronic health conditions on early child development. Journal of Pediatric Psychology, 48(10), 799–811. 10.1093/jpepsy/jsad025 37105227

[18] Field, M., & Lewis, H. (2025). Strengthening Professional Collaboration and Expertise: Implementing and Sustaining a Hospital School Community of Practice. Continuity in Education, 6(1), 91–103. 10.5334/cie.165 40656935 PMC12247828

[19] Forrest, C. B., Bevans, K. B., Riley, A. W., Crespo, R., & Louis, T. A. (2011). School outcomes of children with special health care needs. Pediatrics, 128(2), 303–312. 10.1542/peds.2010-3347 21788226 PMC3387854

[20] Hen, M., & Gilan-Shochat, M. (2022). Exploring the Unique Professional Identity of Hospital Teachers. Continuity in Education, 3(1), 115–126. 10.5334/cie.46 38774286 PMC11104386

[21] Hillier, S. L., Civetta, L., & Pridham, L. A. (2010). Systematic review of collaborative models for health and education professionals working in school settings and implications for training. Educational Health, 23(3), 393. 10.4103/1357-6283.101475 21290358

[22] Hopkins, L., Green, J., Henry, J., Edwards, B., & Wong, S. (2014). Staying engaged: The role of teachers and schools in keeping young people with health conditions engaged in education. The Australian Educational Researcher, 41(1), 25–41. 10.1007/s13384-013-0096-x

[23] Jacob, S. A., & Furgerson, S. P. (2012). Writing Interview Protocols and Conducting Interviews: Tips for Students New to the Field of Qualitative Research. The Qualitative Report, 17(42), 1–10. 10.46743/2160-3715/2012.1718

[24] Jay, M. A., Zylbersztejn, A., Herlitz, L., Deighton, J., Gilbert, R., & Blackburn, R. (2025). Chronic health conditions and school absence, exclusions, and non-enrolment: a cohort study using the Education and Child Health Insights from Linked Data database. Journal of Public Health, 47(3), 414–422. 10.1093/pubmed/fdaf064 40455597 PMC12395948

[25] Jiliberto, F., & Zárate Alva, N. (2025). Influencing Factors In-Hospital School Education: Exploring the Context from the Teacher’s Perspective. Continuity in Education, 6(1), 1–21. 10.5334/cie.126 39897589 PMC11784520

[26] Kanizsa, S. (2024). Luci e ombre dell’alleanza terapeutica nelle pediatrie. Journal of Health Care Education in Practice, 6(1), 17–25. 10.25430/pupj-jhcep-2024-1-3

[27] Karam, M., Brault, I., Van Durme, T., & Macq, J. (2018). Comparing interprofessional and interorganizational collaboration in health international care: A systematic review of the qualitative research. Journal of Nursing Studies, 79, 70–83. 10.1016/j.ijnurstu.2017.11.002 29202313

[28] Keehan, S. (2021). Continuing education in Irish hospital schools: Provision for and challenges for teachers. Continuity in Education, 2(1), 42–59. 10.5334/cie.25 38774896 PMC11104316

[29] LeHo Project. (2015). The institutional environments of home and hospital education (HHE) in Europe. http://www.lehoproject.eu/jdownloads/Public/International%20community/LeHo_-_Institutional_environments_of_HHE_in_Europe_June_2015_0.pdf

[30] Lum, A., Wakefield, C. E., Donnan, B., Burns, M. A., Fardell, J. E., & Marshall, G. M. (2017). Understanding the school experiences of children and adolescents with serious chronic illness: A systematic meta-review. Child: Care, Health and Development, 43(5), 645–662. 10.1136/bmj.m4290 28543609

[31] McNamara, F. (2024). The professional development needs of hospital teachers in Ireland: An exploratory case study. Continuity in Education, 5(1), 50. 10.5334/cie.123 38774598 PMC11104299

[32] Ministero dell’Istruzione, dell’Università e della Ricerca. (2019). Linee di indirizzo nazionali sulla scuola in ospedale e l’istruzione domiciliare [National guidelines for school in hospital and home instruction]. https://www.mim.gov.it/-/linee-di-indirizzo-nazionali-sulla-scuola-in-ospedale-e-l-istruzione-domiciliare

[33] Reeves, S., Pelone, F., Harrison, R., Goldman, J., & Zwarenstein, M. (2017). Interprofessional collaboration to improve professional practice and healthcare outcomes. Cochrane Database Systematic Reviews, 6(6), CD000072. 10.1002/14651858.CD000072.pub3 PMC6481564 28639262

[34] Shaw, S. R., & Brown, M. B. (2011). Keeping pace with changes in health care: Expanding educational and medical collaboration. Journal of Educational & Psychological Consultation, 21(2), 79–87. 10.1080/10474412.2011.571549

[35] Shaw, S. R., & McCabe, P. C. (2008). Hospital-to-school transition for children with chronic illness: Meeting the new challenges of an evolving healthcare system. Psychology in the Schools, 45(1), 74–87. DOI: 10.1002/pits.20280

[36] St Leger, P. (2012). Practice of supporting young people with chronic health conditions in hospital and schools. International Journal of Inclusive Education, 18(3), 253–269. 10.1080/13603116.2012.679320

[37] Steinke, S. M., Elam, M., Irwin, M. K., Sexton, K., & McGraw, A. (2016). Pediatric hospital school programming: An examination of educational services for students who are hospitalised. Physical Disabilities: Education and Related Services, 35(1), 28–45. 10.14434/pders.v35i1.20896

[38] Sullivan, N. A., Fulmer, D. L., & Zigmond, N. (2001). School: The normalizing factor for children with childhood leukemia; perspectives of young survivors and their parents. Preventing School Failure: Alternative Education for Children and Youth, 46, 4–13. 10.1080/10459880109603338

[39] Supper, I., Catala, O., Lustman, M., Chemla, C., Bourgueil, Y., & Letrilliart, L. (2015). Interprofessional collaboration in primary health care: A review of facilitators and barriers perceived by involved actors. Journal of Public Health, 37(4), 716–727. 10.1093/pubmed/fdu102 25525194

[40] Tomberli, L., & Ciucci, E. (2021). Sense of school belonging and pediatric illness: A scoping review. Continuity in Education, 2, 121–134. 10.5334/cie.32 38774888 PMC11104300

[41] Tondi, F. (2022). La scuola ospedaliera nel progetto di “cura” integrale. In V. Boffo (Ed.), La scuola in ospedale (pp. 101–113). Editpress.

[42] The United Nations. (1948). Universal declaration of human rights. The United Nations. https://www.un.org/en/about-us/universal-declaration-of-human-rights

[43] Wei, H. Horns, P., Sears, S. F., Huang, K., Smith, C. M., & Wei, T. L. (2022) A systematic meta-review of systematic reviews about interprofessional collaboration: facilitators, barriers, and outcomes. Journal of Interprofessional Care, 36(5), 735–749, 10.1080/13561820.2021.1973975 35129041

[44] Wikel, K., & Markelz, A. M. (2023). Chronic Health Conditions, School Attendance, and Socioeconomic Factors: A Literature Re-view. The Journal of Special Education Apprenticeship, 12(2), 9. 10.58729/2167-3454.1173

[45] Willig, C., & Rogers, W. S. (Eds.) (2017). The SAGE handbook of qualitative research in psychology. SAGE. 10.4135/9781526405555

[46] World Health Organization. (2010). Framework for action on interprofessional education & collaborative practice. 21174039

