# Supplementary material for: The Hospital School from the Health Professionals’ Perspective: Roles and Collaboration
Source: Contin Educ. 2026 Mar 16;7(1):25–39. doi: 10.5334/cie.273 (PMC13004064; doi:10.5334/cie.273)
Supplement: Supplementary File 1. — Appendix A – Interview protocol. [file cie-7-1-273-s1.pdf]

The Hospital School from the Health Professionals' Perspective: Roles and Collaboration

Supplementary Material 1 – Appendix A: Interview protocol

Dagnino F.M., Caruso, G.P., Dalla Mutta, E. Fante, C. Benigno, V.

Protocol of the interview carried out with six health professionals to explore the perception of Hospital School (HS) and interactions between health professional (HP) staff and HS teachers.

Introduction

1. Introduce yourself and the project

Good…
Thank you for agreeing to do this interview. I'm [NAME OF THE INTERVIEWER], I'm a [ROLE IN THE INSTITUTION] at the Institute for Educational Technology of the National Research Council of Italy and I work with the research team of the Clipso project, which has the aim of fostering the creation of hybrid learning environments (HIC) for hospitalized, sick, or homebound students (SiHo). In the context of the project, we are trying to investigate how Hospital School and Hospital School teachers are perceived by health professionals, this is the reason why we invited you for this interview.

2. Verify that the HP has signed the informed consent form beforehand and agrees to the call being recorded. [Wait for the answer and then start the recordings].

3. Introduce the questions

I will ask you some questions on the topic, but first I' would ask you about your job.

Questions

A. Job information
   a. *What is your job (e.g. psychology, nurse, etc.)*
   b. *In which ward do you work?*
   c. *How long have you been working in that ward?*
   d. *Which patient do you have?*

B. Role of the HS in the care process
   a. *What do you think of the Hospital School Service?*
   b. *In your opinion, what role does the Service play during the hospitalization for children?*
   c. *In your opinion, is the hospital teacher part of the treatment programme?*

C. Relationships between HPs and HS teachers
   a. *What relations do you have with hospital teachers?*
   b. *How are hospital teachers integrated in your ward (e.g.: what kind of relations are there and with whom? Are they formal or informal? Do they participate in team meetings, or do you think they should be part of the team)?*
   c. *What critical issues a teacher may encounter in your ward (or hospital)*
   d. *What role do you (or other health professionals) have or could you have to support the work of the teacher (e.g.: communication about illness)*
   e. *What could, in your opinion, improve the service?*
   f. *Have you experienced any difficulties with regard to the presence of teachers in the hospital?*

Conclusive remarks

1. When the interview ends, ask the interviewee if he/she has any question or remark
2. Thank the interviewee for the time dedicated to the interview and the interesting perspective provided

This document contains supplementary material for the above-mentioned article, as provided by the authors.

The original article can be downloaded from https://doi.org/10.5334/cie.273
