# Supplementary material for: The Hospital School from the Health Professionals’ Perspective: Roles and Collaboration
Source: Contin Educ. 2026 Mar 16;7(1):25–39. doi: 10.5334/cie.273 (PMC13004064; doi:10.5334/cie.273)
Supplement: Supplementary File 2. — Appendix B – Codebooks. [file cie-7-1-273-s2.pdf]

CiE

The Hospital School from the Health Professionals' Perspective: Roles and Collaboration

Supplementary Material 1 – Appendix B: Codebooks

Dagnino F.M., Caruso, G.P., Dalla Mutta, E. Fante, C. Benigno, V.

Tables B1 and B2 present the codebooks for the interviews and the focus group, respectively. The tables report codes/subcodes (second column) aggregated in themes (first column), and the definitions for each code/subcode (third column). Interviews were conducted with six health professionals, and the focus group included seven health professionals.

Table B1

Interviews codebook

Themes	Code/subcode	Definition
Function of HS	Adjectivisation of the HS	HPs use a series of adjectives to define the Hospital School.
	HS role_Combats loneliness and apathy	HPs argue that the HS plays an important role in combating loneliness and other related feelings such as apathy or withdrawal due to illness and hospitalization.
	HS role_Ensures continuity	HPs argue that the HS is a service that, despite the illness, allows for continuity with school.
	HS role_Anchor to the outside world	HPs argue that the HS plays an important role in mediating with the outside world, particularly in maintaining continuity with classmates.
	HS role_Not essential for short hospitalizations	HPs argue that activating the HS is not essential for short hospital stays.
	HS role_Necessary for long stays	HPs argue that activating the HS is necessary for long hospital stays.
	HS role_Ensures normality	HPs argue that the HS anchors the hospitalized student to normality.
	HS role_Bridge for returning to school	HPs state that the HS supports the child in maintaining contact with their home school.
	HS role_Keeps the healthy part of students active	HPs argue that the HS allows students to keep active the resources and abilities that have not been compromised by the illness.

	HS role_Increases receptivity to medical therapies	HPs argue that the presence of the HS is a protective factor also against the illness.
	HS role_Guarantees the right to education	HPs argue that the HS guarantees the right to education.
	HS role_Promotes sociality within the hospital	HPs argue that the HS activities promote socialization among patients.
	HS teacher as an affective figure	HPs highlight the importance of the HS teacher's figure, also as an affective reference.
The HS in the care program	Difficulties in teamwork even among healthcare staff	HPs highlight problems (time, adherence, etc.) in teamwork even among themselves.
	Doubt/teachers' presence not necessary in healthcare teamwork	HPs express doubts about the appropriateness of the teacher's presence during team meetings.
	The role of the hospital teacher_Complementary to the care plan	HPs believe that the teachers' work is complementary to the care of the young patient, like a parallel path.
Teachers' difficulties in the hospital context	Logistical_moving between wards to find students	HPs argue that a difficulty for teachers is related to their continuous moving between different wards.
	Logistical_difficulties in managing spaces	HPs argue that the management of spaces creates difficulties.
	Connected to the illness	HPs argue that teachers can have difficulties in relation to the kids' illness.
	Integration with healthcare staff_problems connected to the interruption of educational activity by healthcare staff	HPs, in recognizing the importance of medical care during the morning hours, also perceive it as an obstacle to the teacher's work.
	Emotional/Relational	HPs report that the relationship with patients and parents can sometimes be tiring because they are not available or are aggressive.
	Privacy_communication problems about the diagnosis	HPs argue that communicating the diagnosis to teachers is not possible for privacy-related reasons.

This document contains supplementary material for the above-mentioned article, as provided by the authors.

The original article can be downloaded from https://doi.org/10.5334/cie.273

	Privacy_opportunity to communicate the "student's functioning" to support teaching	HPs argue that it is necessary for teachers to have information on the student's state of functioning (resources) and on the impact that the illness has on "normal" functioning.
Relations between healthcare professional and HS teachers	Teacher behaviors_positive personal dimensions	HPs report the positivity of some personality traits of the teachers, such as kindness and good manners, as factors that favor the relationship with healthcare staff.
	Teacher behaviors_perceived as inadequate	Healthcare staff talk about behaviors not appropriate for the hospital context on the part of teachers.
	Teacher behaviors_perceived as adequate	HPs talk about appropriate and respectful behaviors of the context on the part of teachers.
	Teacher behaviors_teacher's flexibility	HPs report that a characteristic of the hospital teacher must be flexibility, considering the hospital work context.
	Teachers in the ward_positive exchange with teachers met frequently	HPs report that the constant presence of teachers in the ward favors a positive interaction.
	Itinerant teachers_formal relationship	HPs argue that with the teachers who frequent the ward, there is a formal relationship.
	Itinerant teachers_need to know the teachers who pass through the ward	HPs argue the importance of knowing the teachers who enter the ward anyway.
	Lack of HS/hospital integration_poor knowledge of the school service by healthcare professionals	From the narrative of the HPs, their lack of knowledge of the HS organization emerges.
	Lack of hs/hospital integration_poor collaboration between doctors and teachers	HPs report poor collaboration of medical staff with HS teachers.
	Lack of HS/hospital integration_difficulties connected to the HS presence	Healthcare staff argue that the co-presence of many teachers moving around the wards during the morning hours is a source of chaos.

	HS as a source of information for healthcare staff	HPs refer that teachers are a useful source of information

Table B2

Focus group codebook.

Themes	Code/subcode	Definition
The HS in the care program	Not present in the care program	The HPs point out that the HS is not yet part of the care program.
	Lack of integration	HPs highlight the lack of integration between the HS and the hospital institution.
Teachers' difficulties in the hospital context	Parallelism between the stress of professionals and teachers	HPs point out an overlap between research on stress factors for healthcare professionals and for teachers.
	Fragmentation of hospital work and inadequate spaces	HPs highlight fragmentation of care activity and inadequate spaces.
	Communication difficulties	HPs attribute communication difficulties to the organization of hospital work.
	Lack of staff to act as support with the HS	HPs identify the presence/absence of support figures as a factor influencing the HS
	Fragmentation of teaching due to care commitments	HPs emphasizes the difficulty related to the fragmentation of teaching activity due to the overlap with care activities.
	Privacy difficulties even among professionals	HPs argue that issues related to privacy are critical even among professionals.
	Problem of sharing information related to the multiplicity of teachers	HPs argue that the large number of teachers present in the ward can be an obstacle to the direct communication of information concerning the student.
	Boundaries of privacy	HPs argue for the appropriateness of communicating to the teacher some

		aspects of the patient's functioning in relation to their institutional needs.
Relations between healthcare professional and HS teachers	Lack of communication on the HS organization	The HPs emphasize the difficulty related to the lack of communication of teachers' presence and schedules.
	Need to acquire information on the HS organization	HPs state that they feel the need to acquire more information on the organization of the HS.
Solutions to support the HS teachers' work	Importance of the institution's commitment to the HS	HPs highlight the need for an institutional commitment to support the work of the hospital teacher.
	Need for solutions for individual wards (actions of healthcare staff that support HS) tailoring solutions for individual wards	HPs highlight the need for solutions tailored to individual wards to support the work of the hospital teacher.
	Present HS as part of the care path	HPs talk about the importance of presenting the HS as part of the care path.
	Integration possible only in some wards	HPs point out that not all wards have structured care paths because they do not have long-term patients.
	Need for intermediary figures	HP highlights the need to find intermediary figures.
	Intermediary figures: Psychologist, head nurse, rehabilitation therapist, teacher in the ward	The HPs identify the [name of the profession] as a possible intermediary figure.
	HS for medium and long stays	HPs suggest dedicating HS resources to patients with medium and long stays.
	Desire to overcome the fragmentation of hospital work	HPs argue it is necessary to work to overcome the fragmentation of work typical of the hospital context.
	Educator as support for the teacher's work	HPs identify the ward educator as a support figure for the HS teacher.
Solutions to foster knowledge	Create training paths for teachers in relation to the wards	HPs argue that training is necessary to support them in their knowledge of

		the wards, also in relation to the type of pathology.
	Schedule meetings for mutual acquaintance	HPs consider it useful to start or establish moments of presentation and mutual acquaintance.
	Need for a formal meeting at the beginning of the year	HPs argue the need for an initial meeting at the beginning of the year to get to know each other.
Solutions to foster the exchange of information	Healthcare staff support the need to formally establish periodic meetings	HPs argue the need to formally establish periodic meetings.
	Exchange of information_need to inform the teacher about the student's functioning	HPs believe that knowledge of the student's health status is appropriate in order to be able to personalize the educational intervention.
	Exchange of information_need to share objectives while respecting roles	HPs believe that knowledge of the student's health status is appropriate while maintaining respective roles.
	Exchange of information_need for a coordinating figure in the ward among teachers	HPs state that a figure who mediates information could be supportive of the overall care process of the student.
	Exchange of information_handle the exchange of information with care	HPs believe that it is appropriate to handle the exchange of information with teachers about the student's health status with care to prevent it from negatively influencing their work.
